# Comparison of viscoelastic properties of breast cancer and normal cells using AFM, FLIM and ToF-SIMS techniques

**DOI:** 10.1038/s41598-025-31162-3

**Published:** 2025-12-17

**Authors:** Liubov Shimolina, Yuri M. Efremov, Alexander Gulin, Nadezhda Ignatova, Arseny Aybush, Marina K. Kuimova, Peter S. Timashev, Marina Shirmanova

**Affiliations:** 1 Institute of Experimental Oncology and Biomedical Technologies, Privolzhsky Research Medical University, Minin and Pozharsky Square, 10/1, Nizhny Novgorod, 603005 Russia; 2 Institute for Regenerative Medicine, Sechenov First Moscow State Medical University (Sechenov University), Trubetskaya str., 8/2, Moscow, 119992 Russia; 3 N.N. Semenov Federal Research Center for Chemical Physics, Russian Academy of Sciences, Kosygina Str., 4, Moscow, 119991 Russia; 4 Department of Chemistry, Imperial College London, White City Campus, London, W12 0BZ UK

**Keywords:** Biological techniques, Biophysics, Cancer, Cell biology

## Abstract

The modifications in biomechanical properties of cells and tissue are important in cancer progression, including its different aspects, e.g. invasion, migration, adhesion, signaling, interactions with microenvironment, and immune response. However, our understanding of the changes in physical characteristics of cells, and especially the molecular basis for these changes, caused by malignant transformation remains rather limited. While the differences in stiffness and viscoelasticity of tumor and normal cells have been well documented at the cellular scale, subcellular and molecular alterations have been poorly characterised. In our work, we investigated the stiffness and viscoelastic parameters, mainly determined by the actin cortex, and the microviscosity of the plasma membrane, mainly determined by its lipid profile, in normal and cancer cells. The mechanical properties of the cells were assessed using atomic force microscopy (AFM). The microviscosity of the membrane was visualized by fluorescence lifetime imaging microscopy (FLIM) with the viscosity-sensitive probe BODIPY2. Chemical analysis of cell membranes was performed by secondary ion time-of-flight mass spectrometry (ToF-SIMS). The MCF-10 A (normal epithelial cells) and MCF-7 (human breast cancer) cell lines were used in the study. It was shown that cancer cells were more deformable due to a less organized and more isotropic filamentous structure of the actin cytoskeleton. At the same time they had more viscous plasma membranes, compared to normal cells, in both in vitro and tissue conditions. In the membrane lipid profile, increased signals of sphingomyelin and saturated fatty acids and decreased signals of polyunsaturated fatty acids were detected in cancer cells, which explain the higher microviscosity of their membranes. The obtained data indicate a complex reorganization of cell biomechanics at the cellular, subcellular and molecular levels during malignant transformation, which is important for better understanding of fundamental mechanisms of tumor development.

**Supplementary Information:**

The online version contains supplementary material available at 10.1038/s41598-025-31162-3.

## Introduction

Mechanical properties of cells and tissues play an important role in many physiological processes, including cell adhesion and migration, interaction with extracellular matrix, embryogenesis, wound healing, immune response, etc. Transformation of normal cells into malignant ones substantially alters mechanical parameters at both cellular and tissue levels, which influence biological behavior of tumor cells.

Among different mechanical properties, such as cellular stiffness, viscosity, tension, adhesion force, hydrostatic and osmotic pressure, and shear stress, stiffness is more extensively studied in the context of cancer^1^. Numerous studies demonstrate that, at the level of individual cells, cancer cells have increased deformability and are mechanically softer than their normal counterparts. This was shown, for example, for breast, ovarian, bladder, lung, cervical cancers, metastatic melanoma, and others, using both standard cell lines and patient-derived cultures^2^. Viscoelastic properties of cells are largely determined by the actomyosin cytoskeleton. Normal cells typically have a more organized cytoskeleton^3,4^, which contributes to their stiffness, allowing them to maintain shape and integrity while also enabling some fluid-like responses to mechanical stresses. Softening of cancer cells is mainly the result of remodeling of the cytoskeleton, which include disorganisation of the actin filaments, their depolymerization, and increased ratio of G/F actin^5^. These changes enhance aggressiveness of cancer cells and their invasive and migratory potential.

Besides cellular level viscoelasticity, cancer cells undergo changes at the level of subcellular structures. Specifically, the physical state of plasma membranes differ in cancer and normal cells. Microviscosity is a key physical parameter that characterizes the fluid state of the lipid bilayer, which is important for maintaining cell integrity and function^6^. While higher fluidity is commonly reported for cancer cells compared to normal cells^7,8^, some recent studies have shown the opposite trend — the more viscous membrane in certain types of cancer compared to normal cells^9,10^. This contradiction may arise from differences in cancer type, models or methodological approaches. In general, in terms of comparison of cancer and normal cells, microviscosity is significantly less investigated than other mechanical properties.

It is suggested that the differences in microviscosity of the membranes are due to differences in their lipid composition. Decreased cholesterol and long-chain ceramides and increased level of polyunsaturated fatty acids reduce membrane microviscosity and promote cancer cell motility and metastasis^11^. Higher viscosity of cancer cells’ membranes observed in some studies is attributed to the increased content of saturated fatty acid chains in phospholipids^12^. Therefore, the current consensus is that the membrane fluid state in cancer is altered due to remodelled lipid composition and dynamics in the way that support certain malignant behaviors, but further research is needed to identify these alterations and elucidate their role in different tumor types.

The intracellular actomyosin cortex and the plasma membrane are connected with each other structurally and functionally. The plasma membrane binds the elements of cytoskeleton through transmembrane proteins and electrostatic interactions and mediates the transduction of the external signals that may lead to remodelling of the cytoskeleton^13^. However, the membrane/cytoskeleton interplay has been poorly determined from a biophysical standpoint. It is unclear whether modifications in membrane lipid profile and physical state associated with malignant phenotype correlate with alterations in viscoelastic properties of individual cells.

Our study was designed to better understand the interconnections between plasma membrane microviscosity, membrane lipid profile, and the mechanical properties of cancerous and normal cells. For this, we integrated three powerful techniques within one study - the fluorescence lifetime imaging microscopy (FLIM) to measure microviscosity of plasma membrane in living cells using viscosity sensitive molecular probe, the atomic force microscopy (AFM) to assess the viscoelastic properties of cells with nanoindentation mapping, and time-of-flight secondary ion mass spectrometry (ToF-SIMS) to perform the lipidomic analysis of cell membranes. The study was performed on human breast cancer MCF-7 and normal human mammary epithelial MCF-10 A cell lines, for which biomechanical properties and lipid profile are relatively well characterized by different methods separately, but a comprehensive analysis has not yet been carried out.

## Results

### Mmechanical properties and cytoskeleton of normal and cancer cell lines

**Fig. 1:**
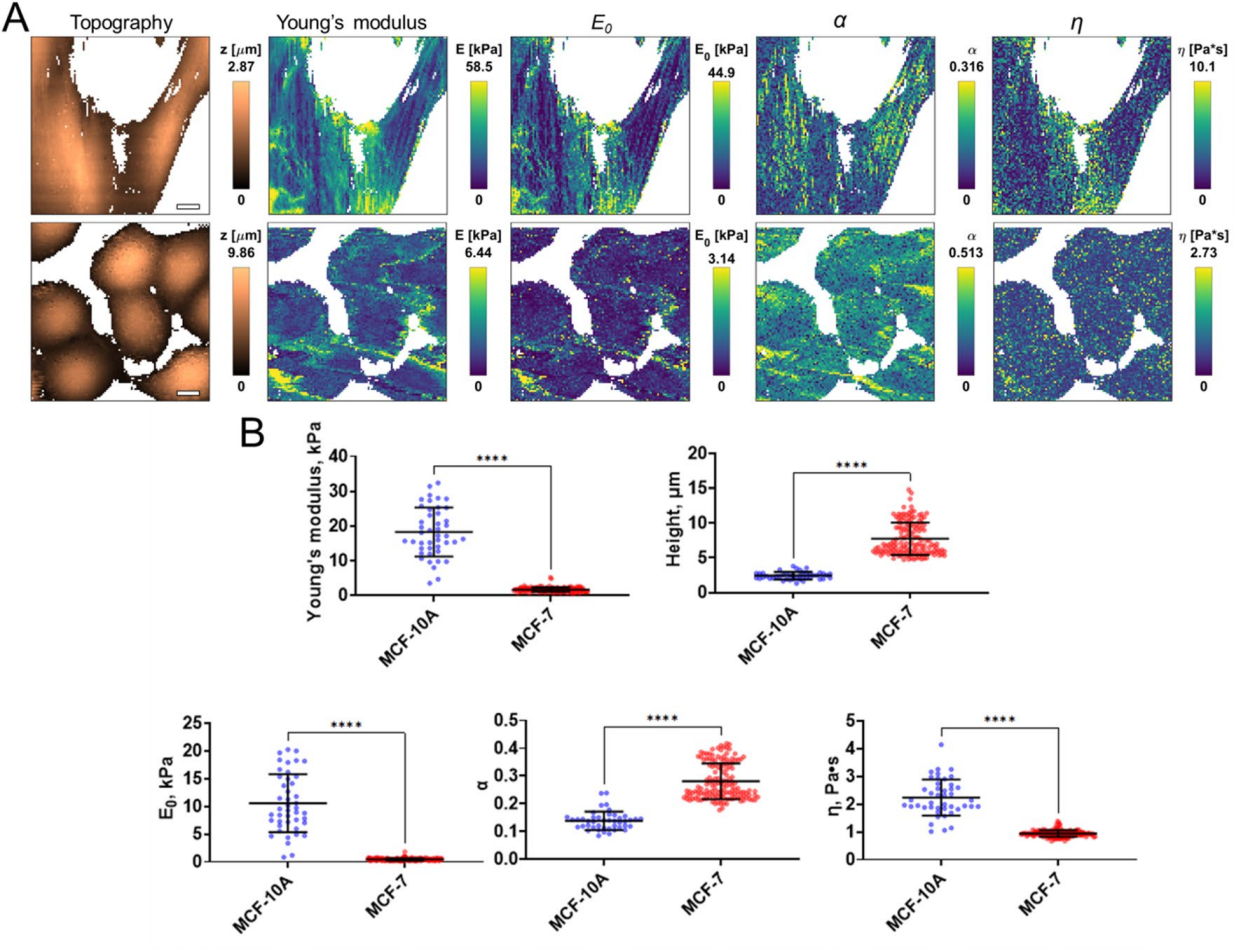
Mechanical parameters of normal epithelial MCF-10 A and breast cancer MCF-7 cell lines assessed by AFM. (**A**) Examples of AFM fast force volume maps, the topographies, the corresponding apparent Young’s modulus and viscoelastic parameters (E0; α; η) maps. Scale bar, 10 μm. (**B**) Quantification of the apparent Young’s modulus and viscoelastic parameters of normal and cancer breast cell lines. Scatter dot plot displaying the measurements for individual cells (at least 30 cells on 10 force volume maps, dots represent a mean value taken from the pixels above the 50% of the maximum height of each cell on map as defined by the corresponding topography images) and the mean ± SD (horizontal lines).

AFM is a gold-standard method for label-free assessing the viscoelastic properties and topography of cells. The apparent Young’s modulus (stiffness), cell height and the viscoelastic parameters (E0, η, and α) were assessed for the normal breast MCF-10 A and cancer MCF-7 cell lines by AFM. The distributions of these parameters taken from fast force volume maps (FFV) are presented in Fig. 1. The MCF-7 cells were substantially higher, while MCF-10 A were spread on the surface, with mean heights of 7.8 ± 2.3 μm vs. 2.4 ± 0.5 μm, *p* < 0.0001. The MCF-7 cells demonstrated drastically lower stiffness than MCF-10 A cells, 1.7 ± 0.7 kPa vs. 18 ± 7 kPa, *p* < 0.0001. Notably, the apparent Young’s modulus depends on the loading rate^14^ due to the viscoelastic nature of cells and does not fully describe cell mechanics, which is why viscoelastic characterization was applied here^15^. The two viscoelastic parameters, scale factor of the relaxation modulus E0 and Newtonian viscosity η, were also significantly smaller for the MCF-7 cells, while the power-law exponent α was significantly larger (0.28 ± 0.06 vs. 0.14 ± 0.03, *p* < 0.0001). This is in agreement with our previous studies, where stiffer cells were shown to have higher values of E0 and lower values of α^16,17^. The obtained data are also consistent with literature data indicating significant differences in the viscoelastic properties of normal and tumor cells^18,19^.

**Fig. 2:**
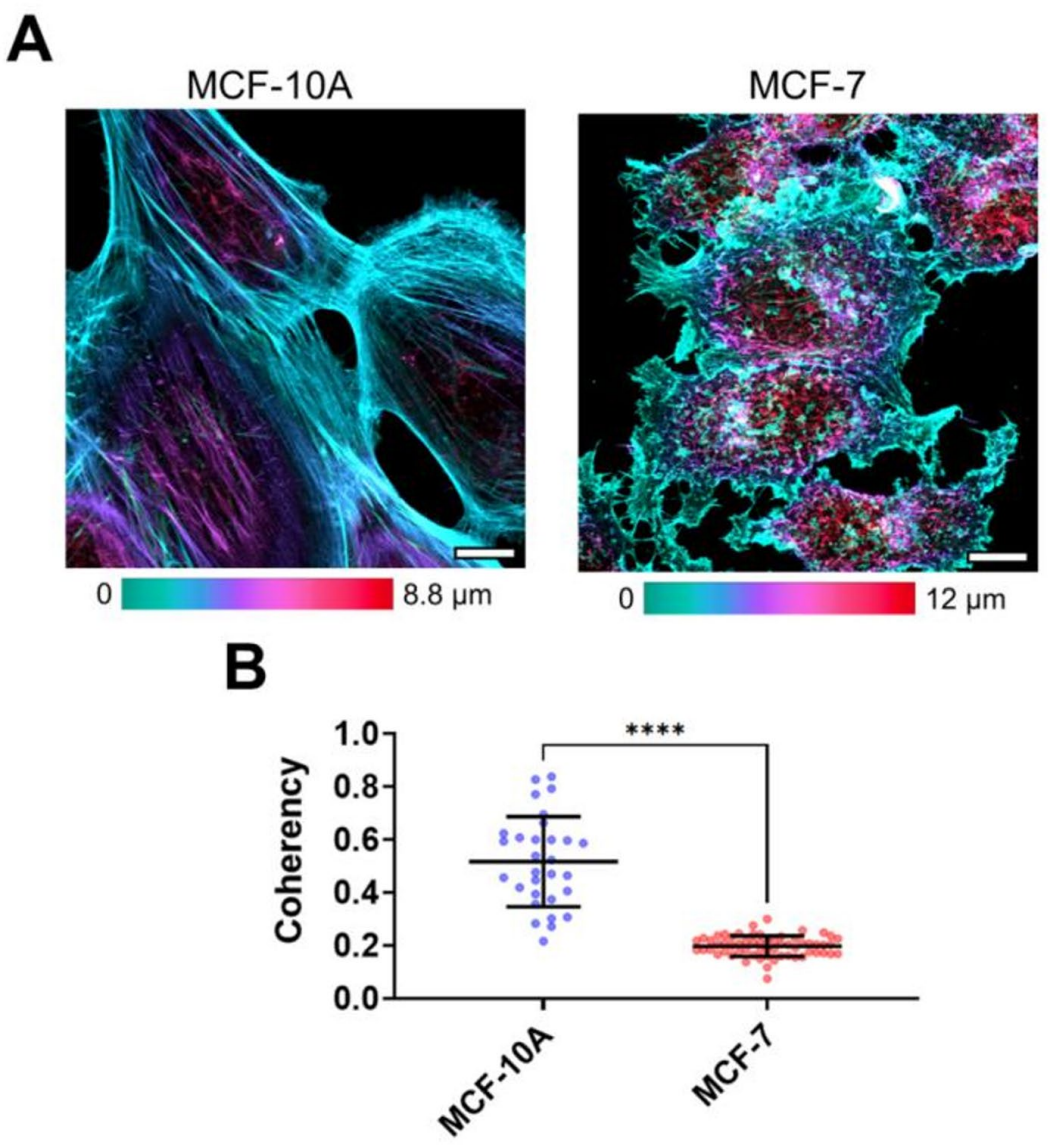
The actin cytoskeleton of normal and cancer breast cell lines by confocal microscopy. (**A**) Color-coded z-projections of the F-actin staining. MCF-10 A cells had a large number of stress fibers, while MCF-7 cells demonstrated irregular shape and membrane ruffles. Scale bar, 10 μm. (**B**) Coherency of the actin cytoskeleton measured in MCF-10 A and MCF-7 cells. MCF-7 cells exhibited a less organized actin network and significantly lower coherency values compared to MCF-10 A cells.

Actin cytoskeleton is known to be a main determinant of cell mechanical properties at the level measured by AFM. It was analyzed by the confocal microscopy in fixed and stained cells (Fig. 2). In agreement with AFM data and previous reports^19,20^, the MCF-10 A and MCF-7 cells had drastically distinctive actin cytoskeleton structure. The benign MCF-10 A cells had well-developed stress fibers located throughout the cells and mostly oriented along its main axis. On the other hand, the cancer MCF-7 cells did not have any stress fibers, were less spread, and had more membrane ruffles. Alignment of actin filaments was quantitatively assessed using the coherency parameter^21^. The actin cytoskeleton exhibited significantly lower coherency in MCF-7 cells (0.20 ± 0.04) compared to MCF-10 A cells (0.52 ± 0.17, *p* < 0.0001), indicating a less organized and more isotropic filamentous structure.

### Microviscosity of plasma membrane in normal and cancer cells

Biomechanics of the cell is determined not only by the elasticity of the cytoskeleton, but also by the viscous properties of the membrane. In this study, we employed FLIM in conjunction with a fluorescent BODIPY2 probe to quantitatively assess the microviscosity of plasma membranes in normal epithelium MCF-10 A and cancerous MCF-7 cell lines. For BODIPY-based viscosity probes, which act as molecular rotors, fluorescence decay time is proportional to the viscosity of the local environment. Upon targeting the plasma membrane, the probe allows direct correlation between measured lifetime (τ) and microviscosity of the lipid bilayer^22^.

**Fig. 3:**
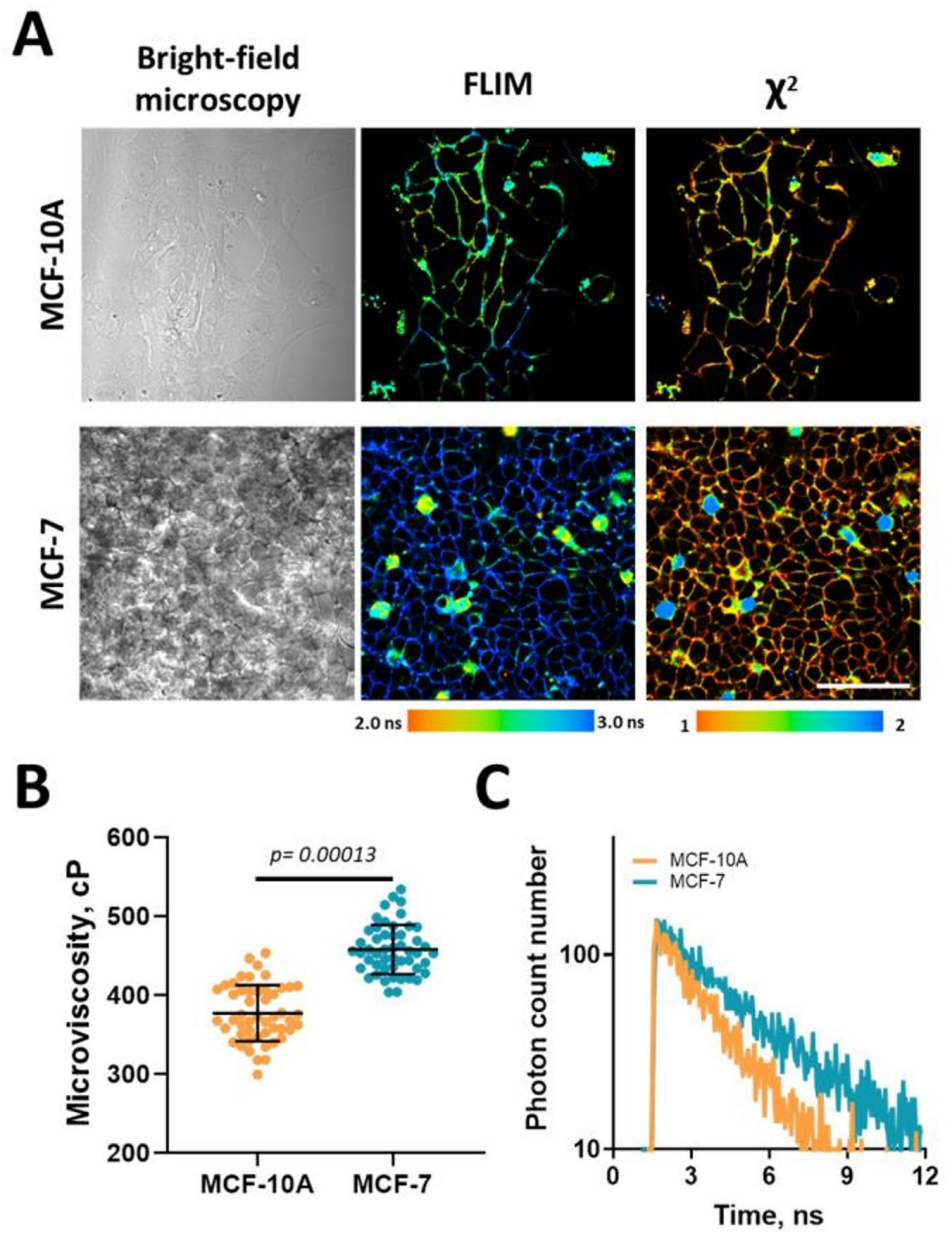
Plasma membrane microviscosity in normal epithelial MCF-10 A and breast cancer MCF-7 cell lines. (**A**) Representative brightfield and FLIM images of cell cultures stained with the BODIPY2 fluorescent molecular rotor. Scale bar: 50 μm. (**B**) Quantification of plasma membrane microviscosity in the individual cells. Mean ± SD, *n* = 60 cells. Dot scatter plot displaying measurements for individual cell membranes (dots) and the mean (horizontal lines) of microviscosity of different cell lines. (**C**) Representative fluorescence decay curves of BODIPY2 in plasma membranes of MCF-10 A and MCF-7 cells.

Figure 3A depicts the effective fluorescent staining of plasma membranes with BODIPY2 in the two examined cell lines. While in cancer cells the probe is retained in the plasma membrane exclusively, in the normal cells it is internalized more quickly and accumulated in the cytoplasm, in addition to the membrane. As follows from the χ^2^ value (the goodness of the fit), the probe had typical mono-exponential fluorescence decay in the membranes, whereas in the cytoplasm this parameter was greater than 1.4, indicating non-monoexponential fluorescence decay of the rotor.

Quantitative analysis of fluorescence lifetimes in the plasma membranes revealed significant differences between the normal and cancer cell lines (Fig. 3B). Normal MCF-10 A cells had a more fluid membrane, with the fluorescence lifetime of the BODIPY2 rotor at 2.49 ± 0.23 ns, corresponding to a microviscosity of 304 ± 56 cP. In contrast, the cancerous MCF-7 cells displayed longer fluorescence lifetime of the rotor, 3.13 ± 0.22 ns, and consequently a higher microviscosity − 483 ± 66 cP (*p* = 0.00013).

**Fig. 4:**
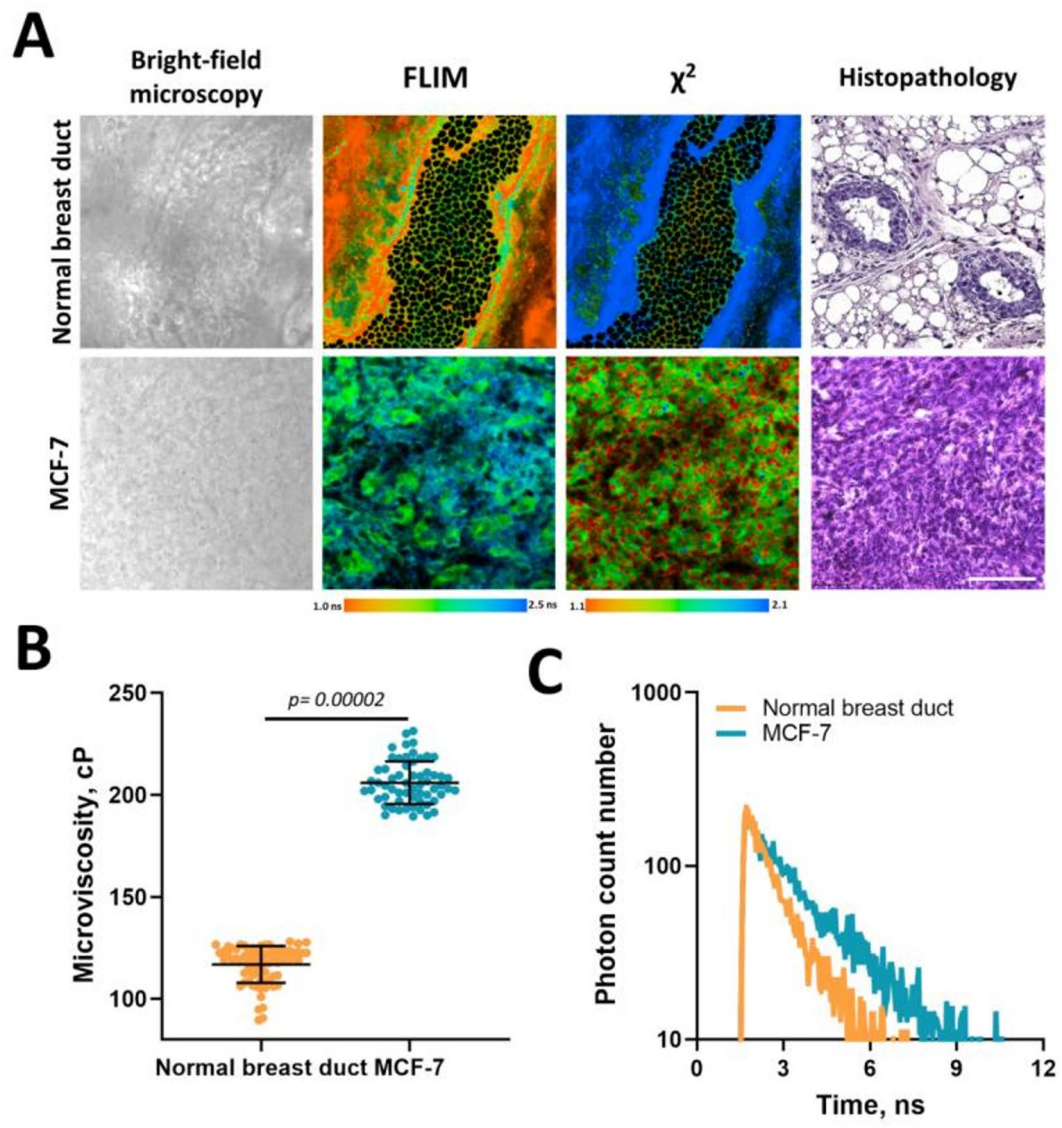
Microviscosity of normal mouse breast tissues and human breast cancer assessed from FLIM with BODIPY2. (**A**) Representative histological, fluorescence intensities and FLIM images of normal and cancer tissue samples stained with the BODIPY2 fluorescent molecular rotor. Scale bar: 60 μm. (**B**) Quantification of microviscosity in the tissues. Mean ± SD, *n* = 50 cells. Dot scatter plot displaying measurements for individual cell membranes (dots) and the mean (horizontal lines) of microviscosity in different samples. Importantly, within the tissue only the regions of interest where the rotor demonstrated a monoexponential fluorescence decay (χ^2^ ≤ 1.40) were analyzed, which indicated its correct operation. (**C**) Representative fluorescence decay curves of BODIPY2 in normal breast duct cells and MCF-7 tumor cells.

To find out whether the microviscosity of normal and tumor cells differs at a more complex tissue level, FLIM with BODIPY2 was performed on the isolated samples of human breast cancer MCF-7 and normal mouse mammary ducts (Fig. 4). Histopathological examination of tumor tissue samples confirmed the presence of cancer cell complexes with large nuclei and weakly basophilic cytoplasm. Mitotic activity was high, especially at the periphery of the nodules, and areas of spontaneous necrosis were insignificant.

In the tissues, the BODIPY2 rotor was distributed diffusely, both in the cytoplasm and in the plasma membranes, in contrast to monolayer cancer cells, where it was localized mainly in the plasma membrane. So that the lifetime values ​​measured in the individual cells within the tissues contained contributions from various cellular structures, including a more fluid intracellular environment, which could be a possible reason for generally faster decay times in the tissues than in the culture. FLIM data analysis showed that in tumor MCF-7 tissue, the fluorescence lifetimes amounted to 2.06 ± 0.05 ns, which corresponded to a viscosity of 208 ± 11 cP. In normal breast duct cells the fluorescence lifetime was lower, 1.55 ± 0.06 ns, which corresponded to a viscosity value of 116 ± 9 cP (Fig. 4) (*p* = 0.0004). Therefore, the obtained tissue data coincide with the tendency that was obtained in in vitro experiments showing the more viscous membranes in the tumor.

In addition, lipophilic in nature, BODIPY2 effectively stained adipose tissue within the mammary glands, but the χ^2^ value (the goodness of the fit) in this localization was greater than 1.5, indicating incorrect rotor operation, possibly involving the aggregation of fluorophore, which compromises its ability to measure viscosity.

Therefore, the results obtained on cell cultures and tissues are consistent with each other and indicate that breast cancer cells have more viscous membrane than normal epithelium.

### Lipid composition of plasma membrane in normal and cancer cells

It is known that the viscosity properties of membranes are largely determined by the qualitative and quantitative lipid composition. ToF-SIMS was used for the lipidomic analysis of cell membranes. This high-resolution surface analysis technique provides detailed molecular and elemental information with exceptional sensitivity bridging the gap between biochemical composition and biophysical state of the cell membrane. In our work, using time-of-flight secondary ion mass spectrometry ToF-SIMS, we register the signal mostly from the outer layer of the plasma membrane, while a signal from the lipids of the inner layer is relatively small.

**Fig. 5:**
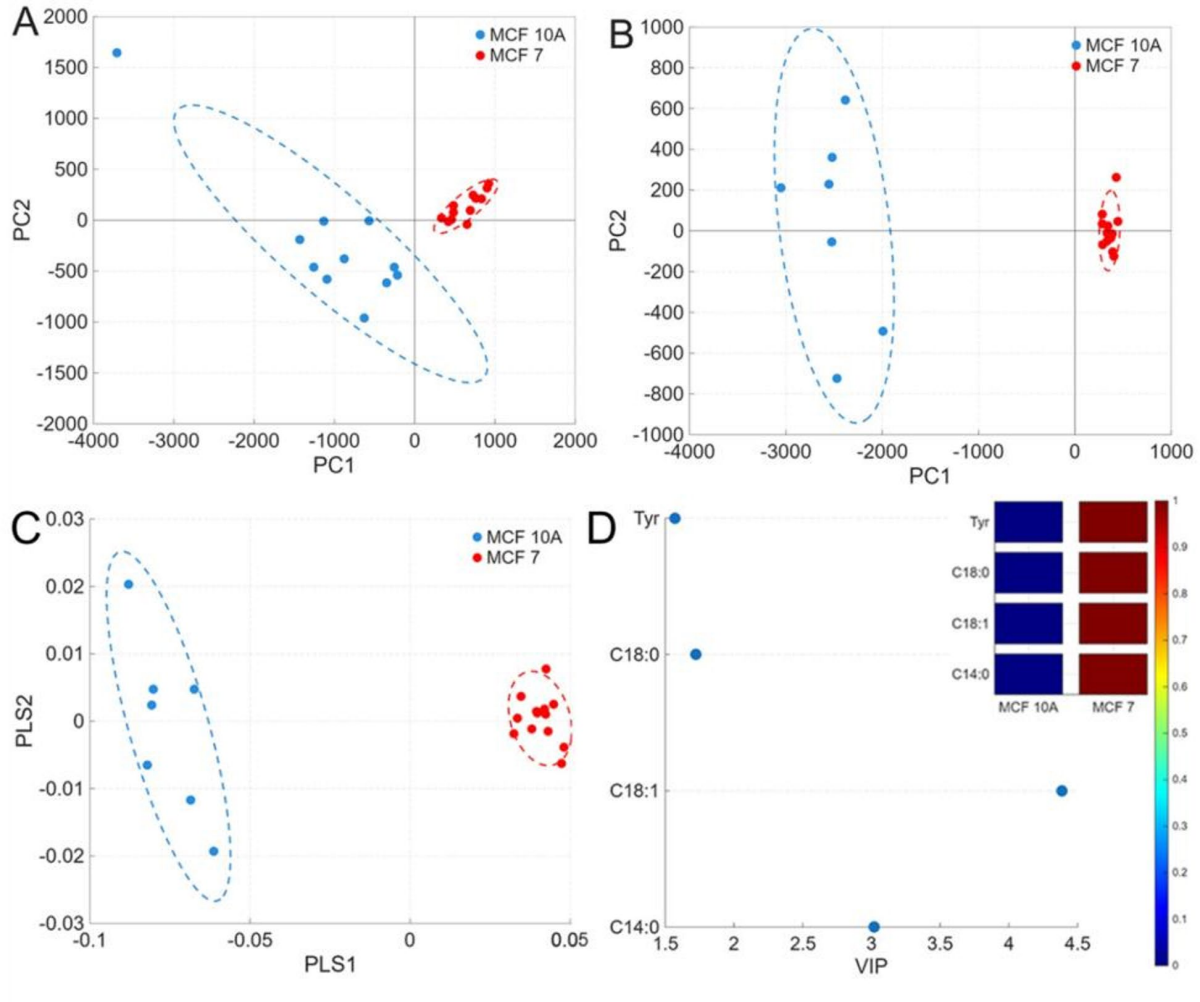
PCA of the composition of normal MCF-10 A and cancer MCF-7 cell samples in positive (**A**) and negative (**B**) ion modes from the ToF-SIMS data. Ellipses indicate 95% confidence intervals. (**C**) PLS-DA score plots of MCF-10 A and MCF-7 cells in negative ion mode. (**D**) VIP scores reveal 4 ions with the VIP values of > 1.0. Graphic visualization of signals with a scale (red - high, blue - low).

First, untargeted membrane lipid profiling was performed to analyze normal MCF-10 A and cancer MCF-7 cell samples. Full ToF-SIMS spectra in both positive and negative ion modes are shown in Fig. S2. It was found using Principal Component Analysis that the MCF-7 and MCF-10 A cell lines have different membrane composition profiles in both positive and negative ions modes (Fig. 5), with a higher variability of mass spectra in the case of normal cells. To identify specific components that reliably differentiate normal and cancer cells Partial Least Squares Discriminant Analysis (PLS-DA) was used, which also demonstrated significant differences in chemical composition of normal and cancer cell membranes (Fig. 5C). In order to measure the importance of variables from the PLS-DA, the VIP (Variable Importance in Projection) score was evaluated. In the peak list of known lipid and amino acids species, the highest VIP scores were obtained in negative ion mode for myristic (С14:0), stearic (С18:0), and oleic (С18:1) fatty acids (Fig. 5D), which are among the most abundant fragments of phospholipids.

**Fig. 6:**
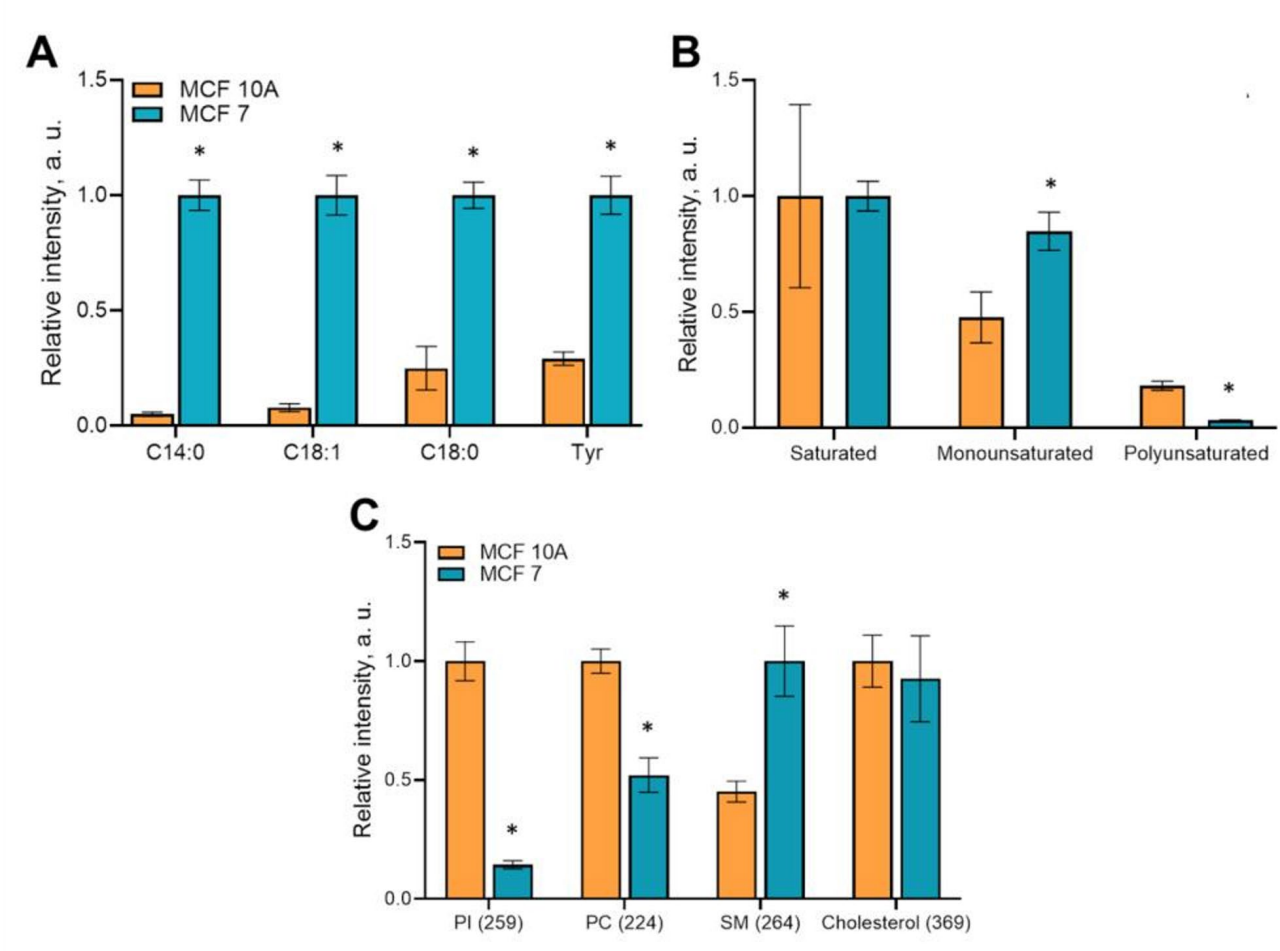
Lipid profile of normal MCF-10 A and cancer MCF-7 cells. (**A**) Relative normalized intensity of these ions. Mean ± SD. *, *p* < 0.005 with normal cell line. (**B**) Fatty acids chains. The signal of unsaturated fatty acids was normalized to signal of saturated fatty acids for each sample. Mean ± SD. *, *p* < 0.005 with normal cell line. (**C**) Lipid content of phosphatidylinositol (PI), phosphatidylcholine (PC), sphingomyelin (SM) and cholesterol. PI ions were obtained in negative ion mode. Mean ± SD. *, *p* < 0.005 with normal cell line.

These three fatty acids showed statistical differences (*p* < 0.0001) in their relative signal intensity between cancer and normal cells (Fig. 6A). Fatty acids (C14:0) and (C18:0) are saturated, which may partially explain the increased membrane microviscosity in cancer. A higher signal of ion with m/z 180.06 was also detected in cancer cells (*p* < 0.0001), which can be attributed to amino acid tyrosine. Tyrosine is known to play an important role in the regulation of membrane viscosity through interactions with transmembrane proteins and lipid rafts.

Figure 6B depicts the difference in fatty acid chains of plasma membrane lipids. MCF-7 cancer cells demonstrated 1.8 times higher content of monounsaturated fatty acids (*p* < 0.0001) and 6 times lower content of the PUFA (*p* < 0.0001) compared to normal cells. A decrease in the PUFA in fatty acid tails can also contribute to increased microviscosity of tumor cell membranes.

Analysis of the specific lipid components essential for viscous membrane state showed that the signal of cholesterol ions (m/z 369.3) was comparable in MCF-10 A and MCF-7 cells, while phospholipids signals were different. The signal of phosphatidylinositol (PI) ions (m/z 259.1) was 7.1-fold lower (*p* < 0.0001), phosphatidylcholine (PC, m/z 224.1) − 2-fold lower (*p* < 0.0001) and sphingomyelin (SM, m/z 264.2) 2.2-fold higher (*p* < 0.0001) in cancer compared to normal cells. It is known that sphingomyelin makes the membrane more viscous, while phosphatidylcholine and phosphatidylinositol liquefy it^23^, which is consistent with our viscosity data.

It should be noted that although trends in lipid yield were clearly visible, quantification may not reflect the true cellular lipid content due to matrix effects^24,25^ and low ion yields for some lipid species. For instance phosphatidylcholines and sphingomyelins had a lot of common fragments and only m/z 224 (C_8_H_19_NPO_4_^+^) and low intensity m/z 264 (C_18_H_34_N^+^) ion could be used to distinguish them^26^. Cholesterol is known to have two specific ions without a hydrogen [M-H]^+^ (m/z 385) or without a hydroxyl group [M-OH]^+^ (m/z 369). Since [M-OH]^+^ had a higher intensity it was utilized for evaluation. However, a recent study showed a 50% increase in the peak intensity ratio of m/z 369 to m/z 385 in cancer tissue compared to normal tissue^27^.

## Discussion

Dynamic change in the mechanical properties of cells is directly associated with transformation of normal cells into cancerous. Owing to extensive modifications in cytoskeletal networks and membrane composition cancer cells realize their key biological functions - invasiveness and metastasis, as they strongly affect interconnections with the extracellular matrix, adhesion and signaling events. These modifications inevitably result in the changes of mechanical properties at the subcellular, cellular and tissue levels. Here, by performing a comparative study of breast cancer and normal human mammary epithelial cells, we found significant differences in plasma membrane microviscosity assessed by FLIM, cell stiffness assessed by AFM, and membrane chemical profile assessed by ToF-SIMS.

The biomechanical properties of tumor cells are largely determined by the elasticity of the cytoskeleton. Overall, in our work, the values of the apparent Young’s modulus (2–20 kPa) and of the viscoelastic parameters (E0 ≈ 0.5–10 kPa; α ≈ 0.14–0.3; η ≈ 0.9–2.2 Pa∙s) are close to those obtained in previous studies on cancer and epithelial cell lines^16,28,29^. The normal breast cell line MCF-10 A was substantially stiffer than colorectal epithelial cancer cell lines measured previously by the same technique^17^. That also agreed with differences in the actin cytoskeleton structure, well-developed actin fibers in normal MCF-10 A cells and disorganized isotropic fibers in cancer MCF-7 cells^21^.

There are many works presenting a direct comparison of mechanical properties between normal and cancerous cells using AFM. A general view is that single cancer cells are more deformable and their viscoelastic parameters are reduced as compared to their normal counterparts^30–33^. This has been shown for different types of cancers including, for example, bladder, prostate, cervix, ovary and breast cancer^31^. AFM was used to evaluate the viscoelastic properties of human breast epithelial cells with different metastatic capabilities in both adherent and suspended states^20^. The obtained data show that the Young’s modulus of adherent cells is inversely correlated with their malignancy and that the location and intensity of F-actin support the mechanical phenotype. Suspended cells were found to exhibit lower elasticity than adherent cells due to the distribution of actin filaments in the cell cortex as well as reduced polymerization^20^. In our study, we found that Young’s modulus was significantly higher in normal MСF-10 A cells, which is consistent with the results of other authors.This reduced stiffness may facilitate invasive potential by enabling easier deformation and migration through dense extracellular matrices and narrow vascular spaces. It is well established that cellular elasticity is strongly linked with the cell cytoskeleton^34–37^. In line with other studies^19,21,38^, our results showed that cancer cells did not develop well-organized filamentous actin structures, as opposed to normal cells. However, the viscoelastic properties of the cells cannot be fully described by the rigidity of the cytoskeleton alone, since they depend on the complex interaction of many factors, including the biochemical and biophysical state of the membrane.

Microviscosity of lipid membranes is a fundamental physical property that determines the permeability of bilayer for diffusing molecules and the functioning of membrane proteins - receptors, channels, transporters, enzymes. During neoplastic transformation, the microviscosity (or fluidity) of cell membranes can change, and these changes can vary depending on the histogenesis of the tumor. Previous data about membrane viscosity (fluidity) of cancer versus normal cells are poor and quite contradictory. In general, cancer cell membranes were reported to be more fluid compared to normal cells. For example, decreased microviscosity of membrane lipids was shown in leukemic cells compared with intact lymphocytes using fluorescence polarization technique^39^. The membranes of human lung cancer tissues were more fluid than those of normal lungs as assessed by electron paramagnetic resonance (EPR) with a lipophilic spin probe^40^. The study by Hattori et al. using EPR showed that the phospholipid membrane of glioma was more fluid than white matter, and more rigid than grey matter^41^. Sherbet et al. found increased membrane fluidity in mouse melanoma B16 and lymphoma L5178 cells using steady-state fluorescence polarization^42^. Campanella et al. showed higher membrane fluidity in human cerebral astrocytoma cells by studying their lipid composition^43^. Kaur et al. used fluorescence polarization with the probe 1,6-diphenylhexatriene (DPH) to investigate the role of membrane fluidity in the carcinogenic transformation of colon epithelial cells. Membrane fluidity was shown to increase during the early stages of chemical induction of carcinogenesis^44^. Some other papers demonstrate the opposite trend. For example, Lee et al., using the сholesterol depletion (methyl-β-cyclodextrin) and lipid raft isolation method, found that breast cancer cell lines MCF-7 and MDA-MB-231 contained more lipid rafts and had higher membrane viscosity than their normal counterparts PZ-HPV7 and MCF-10A^9^. In the work of Heydarian et al., by adding nanomagnetic particles to the cell culture medium and using magnetic tweezers technique to perform creep testing, it was shown that MCF-7 has higher gel point frequency, thus these cells can be described by more solid compared to their corresponding healthy cells^10^. In our recent work, we correlated the cell stiffness measured by AFM with the membrane fluidity in the series of colorectal cancer cell lines with varying migratory activity and found the positive correlation between the Young’s modulus and plasma membrane viscosity. This positive correlation could be in part due to the effect of dense cytoskeleton on the membrane viscosity^45^. In the present study, the microviscosity of the plasma membranes of MCF-7 breast cancer cells was higher than that of normal human mammary epithelial cells in both in vitro and tissue conditions. To the best of our knowledge, this is the first time that microviscosity differences between cancer and normal human mammary epithelial cells were identified within the tissue. It is also of note that this trend does not follow the cell stiffness data measured by AFM, i.e. more stiffer normal cell line was found to have lower plasma membrane viscosity. We attribute the apparent contradiction with previously observed correlations between stiffness and microviscosity to malignant transformation itself, rather than to tissue-specific (e.g., breast vs. colon) differences. Specifically, we propose that this transformation entails a coordinated adaptation of mechanical properties: cancer cells develop greater whole-cell deformability alongside a more viscous plasma membrane. Increased membrane viscosity, in turn, may prevent the formation of cell–cell junctions^46,47^ and favor the stabilization of lipid rafts^48,49^. While cellular stiffness and membrane fluidity are interconnected in determining the migratory capacity of cancer cells, these connections are altered upon the transformation of normal cells into cancerous ones, and lipid remodeling becomes the dominating factor. A summary table of the parameters studied is provided in the supplementary information (Supplementary information, Table S1).

**Fig. 7:**
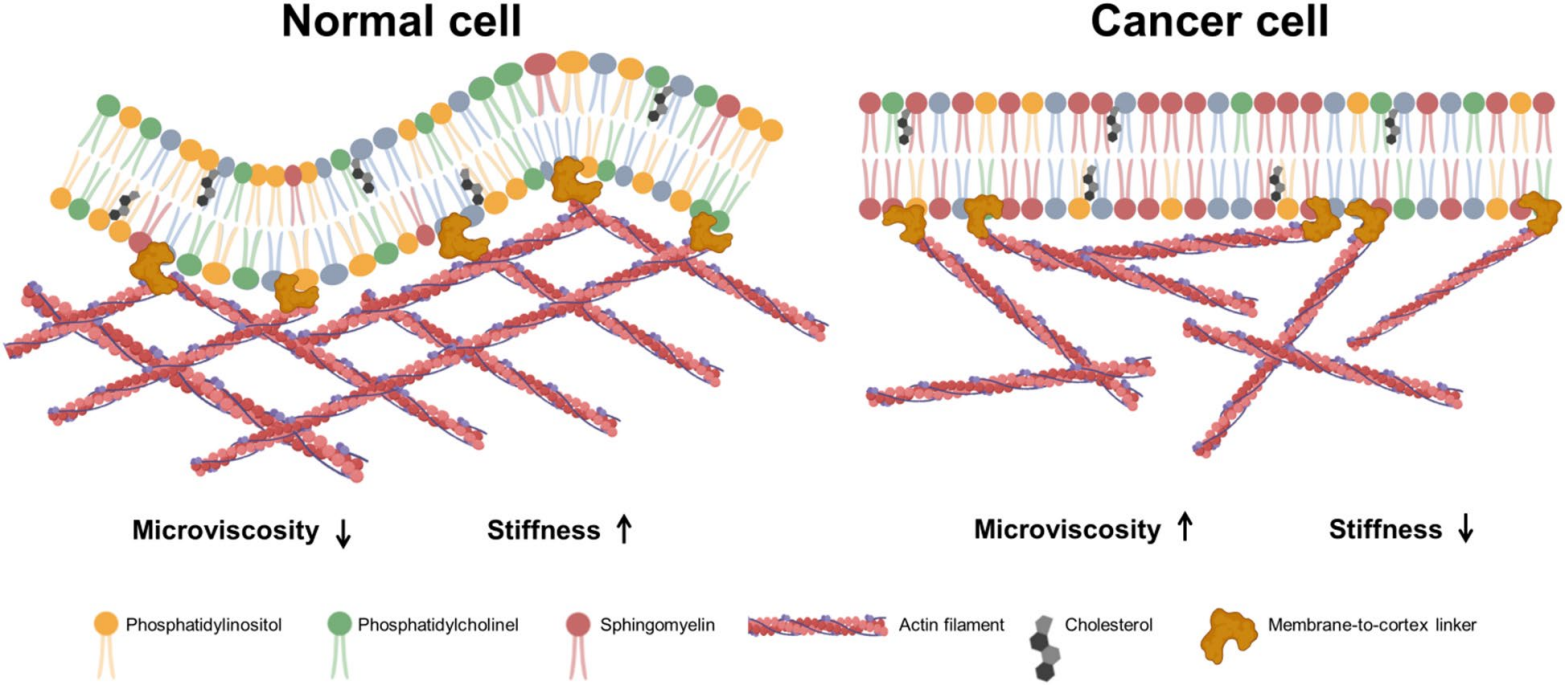
A schematic diagram of changes during neoplastic transformation.

Membrane lipid constituents directly influence membrane biophysics, thereby modulating the activity of membrane proteins serving as ion channels, transporters, receptors, signal transducers, and enzymes—profoundly impacting cellular functionality. Lipidomic dysregulation is strongly implicated in cancer initiation and progression. Substantial evidence indicates significant reorganization of the lipidome in breast cancer cell membranes compared to normal breast epithelium, critically driving tumor progression and metastasis^50^. For example, as principal membrane components, PCs promote cancer cell proliferation, with synthesis augmented by de novo fatty acid production^51^. We found elevated PC signals in MCF-7 tumor cells, aligning with reports of elevated alkyl acyl PCs in malignant breast cells. In the work of Dória et al. elevated levels of alkyl acyl PCs were found in malignant breast cancer cells compared to non-malignant cells^52^. Increased levels of monounsaturated fatty acids (e.g. PE(O-16:00/18:01) and PE(O-18:00/18:01)) were observed in an aggressive mouse breast cancer cell line^52^. Our data revealed a significant increase in monounsaturated fatty acid in MCF-7 membranes. In addition, we also found increased signals of fatty acid residues C14:0 and C18:0 in tumor cell membranes, which is further consistent with the data of other authors. Increased levels of sphingomyelin have also been found in non-malignant normal primary breast cells compared to human breast cancer cell lines^53^. Estrogen-mediated suppression of fatty acid desaturases has been reported for MСF-7 cells. For example, in MCF-7 cells, estrogen caused a dose-dependent decrease in the level of scyadonic acid (5,11,14–20:3, ScA) through a marked inhibition of FADS1 activity^54^. We found a significant reduction in the content of polyunsaturated fatty acids in the membranes. Additionally, evidence demonstrated that sphingomyelin is reduced in a number of cancer cell membranes and that there is a relationship between its levels and tumorigenesis^55^. In breast cancer, especially in ER+ (including MCF-7), an increased content of sphingomyelin was found, which stabilized lipid rafts, enhancing oncogenic signaling and endocrine sensitivity^56^. In addition, sphingomyelin, due to its structure, is able to increase the microviscosity of membranes^57^. Some studies indicate that the two layers of mammalian plasma membranes may differ in lipid content^58,59^. Thus, the outer layer contains more cholesterol and sphingomyelin than the inner layer^58^. In our work, using ToF-SIMS, most of the signal is registered from the outer layer of the plasma membrane. We identified a higher sphingomyelin content in MCF-7 cancer cells than in normal MCF-10 A cells, which can also contribute to the higher microviscosity^60^. Based on the results of this study, we hypothesize that during neoplastic transformation, cells undergo specific lipid remodeling, creating a more ordered, viscous membrane that serves to stabilize signaling platforms (lipid rafts) and diminish cell-cell adhesion. A schematic diagram of changes during neoplastic transformation is shown in Fig. 7.

Amino acids play a key role in the regulation of cell membrane viscosity through interactions with lipids, proteins, and water at the interface. Thus, Tyrosine (Tyr), an aromatic amino acid, plays an important role in the regulation of membrane viscosity through interactions with lipid bilayers, especially transmembrane proteins and lipid rafts. Its aromatic ring participates in π-stacking or cation-π interactions with nearby phospholipid or cholesterol head groups, stabilizing the lipid packing and reducing fluidity^61^. The aromatic ring of Tyr stabilizes lipid rafts by increasing membrane rigidity (similar to the action of cholesterol)^62,63^. Increased amounts of Tyr may be associated with high tyrosine kinase activity, which is known to play a significant role in the signaling pathways of MCF-7 breast cancer cells^63^. In addition, tyrosine kinase regulates the actin-myosin network via Rho-GTPase by polymerizing actin^64^, which may promote the creation of tight complexes under the membrane that restrict lipid movement, thereby increasing viscosity. Therefore, the increased Tyr signal we found in the membranes of MCF-7 tumor cells may also contribute to the increased viscosity.

Our study has potential limitations. One is that the BODIPY2 probe is distributed throughout the tissue without specificity for plasma membranes. As a result, different cellular structures, through binning of signal from neighboring pixels, may contribute to the measured microviscosity values, in addition to plasma membranes, and microviscosity in tissue, both tumor and normal, is lower than in corresponding cultured cells. Lower values of microviscosity in tumor in comparison with cell line were observed previously^65,66^. It is also internalized more quickly by normal epithelial cells than by cancerous cells. To minimize cytoplasmic diffusion, an alternative probe could be considered in future studies. Another limitation is that measurements in tissues were done in the freshly excised samples but not in vivo. This is because mouse mammary duct and the orthotopic breast tumors are not accessible with standard objective lens for intravital investigation. Additionally, the results of this study are based on a single cancer cell line. Including different breast cancer cell lines with varying metastatic potential would strengthen the results, and this will be addressed in future research.

Thus, we conclude that during neoplastic transformation, breast epithelial cells undergo significant biochemical and biophysical changes that affect cell biomechanics. The changes are multidirectional - tumor cells lose their stiffness, which favors migration and invasion, but at the same time maintain a high membrane viscosity due to remodelling of lipids, which stabilizes lipid rafts for signaling and resists fragmentation during migration. We suggest that during neoplastic transformation, tumor cells coordinate low cytoskeletal elasticity (for mechanical penetration) and high membrane viscosity (for the effectiveness of signaling pathways) as complementary survival strategies. Studying cell biomechanics during neoplastic transformation is critically important, since these changes are not just a consequence, but active drivers of oncogenesis, invasion, metastasis and resistance to therapy.

## Conclusion

The neoplastic transformation drives profound biochemical and biophysical alterations that fundamentally reshape cellular biomechanics which is associated with cancer progression. Studying the viscoelastic properties of normal and tumor cells provides critical insights into the biomechanical changes associated with cancer progression. Here, using a combination of multiple techniques - AFM, FLIM microscopy with a BODIPY2 fluorescent molecular rotor, confocal microscopy and ToF-SIMS - we sought to find any differences in the viscoelastic properties of normal MCF-10 A and tumor MCF-7 cells. Our results, along with the existing literature, indicate that tumor cells exhibit reduced stiffness and increased membrane viscosity compared to their normal counterparts. We propose that this coordinated adaptation – low cytoskeletal elasticity enabling mechanical penetration coupled with high membrane viscosity due to lipid remodeling ensuring signaling efficacy and structural integrity – represents a sophisticated, complementary survival strategy. Therefore, understanding the viscoelastic signatures of cancer cells not only expands fundamental knowledge of tumor mechanics but also opens new avenues for diagnostic and therapeutic strategies.

## Methods

### Cell cultures

The MCF-10 A (epithelial cell line derived from benign proliferative breast tissue, ATCC No: CRL-10317) and cancer MCF-7 (a human breast cancer, ATCC No: HTB-22) cell lines were used in the study. The cancer cells were cultured in DMEM containing 100 µg/mL penicillin, 100 µg/mL streptomycin sulfate and 10% fetal bovine serum in the incubator at 37 °C in a humidified atmosphere with 5% CO_2_. The normal cells were cultured in DMEM-F12 containing 100 µg/mL penicillin, 100 µg/mL streptomycin sulfate, 5% horse serum, 10 µg/mL human insulin, 0.5 µg/mL hydrocortisone and 10 ng/mL EGF in the incubator at 37 °C in a humidified atmosphere with 5% CO_2_.

### Samples of normal and tumor tissue

Normal mammary gland tissue samples were collected from Balb/c mice, female, 10 weeks old, weighing 20–25 g. To obtain tumor tissue samples, 10^6^ MCF-7 cells were resuspended in 100 µl of Matrigel and were inoculated subcutaneously into the right thigh of the Nude mice, female, 10 weeks old, weighing 20–25 g. After the tumors reached a size of at least 7 × 8 mm, tumor tissue samples were collected for analysis. Before the procedure, animals were anesthetized by intramuscular injection of a mixture of Zoletil 100 (40 mg/kg, Virbac SA, France) and Rometar (10 mg/kg, Spofa, Czech Republic). Microviscosity visualization was performed immediately after sample collection. All experimental procedures conducted on animals were approved by the Ethical Committee of the Privolzhsky Research Medical University (approval №09 from 30/06/2023). All methods were carried out in accordance with relevant guidelines and regulations.

### Membrane microviscosity measurement via FLIM with a molecular rotor

Membrane microviscosity was assessed using the molecular rotor BODIPY2 (4,4-difluoro-4-bora-3a,4a-diaza-s-indacene), which exhibits fluorescence lifetime sensitivity to local viscosity^67^. This probe is suitable for biological studies due to its dynamic lifetime range and reliable performance in cellular environments^67^.

Cells were seeded in phenol red-free DMEM on glass-bottomed FluoroDishes (Life Technologies, USA) and incubated for 24 h. For staining, cells were washed with ice-cold Ca^2+^/Mg^2+^-free Hank’s solution, incubated at 4 °C for 3 min, and stained with BODIPY2 (4.5 µM in PBS, 0.1% DMSO). Fluorescence Lifetime Imaging Microscopy (FLIM) was performed within ~ 20 min post-staining. Normal and tumor tissue samples were stained in the molecular rotor using a modified protocol: samples were placed in ice-cold BODIPY2 rotor solution (20 µM in PBS, 0.4% DMSO) for 5–10 min. Samples were then washed in PBS and microscopic imaging was performed immediately.

Imaging was performed on a Zeiss LSM 880 microscope equipped with a Becker & Hickl SPC 150 TCSPC FLIM module and a Mai Tai HP femtosecond laser (Spectra Physics; 80 MHz repetition rate, 140 fs pulse width). BODIPY2 was excited via two-photon excitation at 850 nm. Emission was collected within the 500–550 nm range using a 40×/1.2 NA objective. FLIM images were acquired at 1–2% laser power with a 60-second collection time per field to ensure ≥ 5000 photons per decay curve. Ten random fields were imaged per sample.

Fluorescence lifetime analysis was performed using SPCImage 8.3 (Becker & Hickl). Lifetimes were extracted from manually selected plasma membrane regions. Decay curves were fitted to a monoexponential model (χ^2^ = 0.8–1.2), and lifetime values were converted to viscosity (cP) using a previously established calibration curve^68^.

### Mass spectrometry assay via ToF-SIMS

For ToF-SIMS analysis, MCF-10 A and MCF-7 cells were seeded at a density of 5 × 10^5^ cells on poly-L-lysine-coated coverslips in 35 mm m-Dishes (Ibidi, Germany) and cultured in full DMEM medium for 24–48 h (37 °C, 5% CO_2_). Following incubation, cells were washed three times with PBS, fixed with 4% paraformaldehyde (60 min, room temperature), and washed again with PBS and mQ water. Prior to analysis, samples were pre-dried and dried under a gentle argon stream at room temperature. While this sample treatment protocol could possibly lead to an altering of lipid organization and affect membrane integrity, it maximized lipid ion yield compared to other sample preparation techniques^69^.

Chemical profiling and lipid analysis were performed using a ToF-SIMS 5 mass spectrometer (ION-TOF, Germany) with a 30 keV Bi_2_⁺ liquid metal ion gun. Spectra were acquired in both positive and negative polarities from 300 × 300 μm^2^ scan areas with lateral resolution 64 × 64 pixels. The primary ion dose density did not exceed 3.5 × 10^11^ ions/cm^2^ (within the static SIMS limit). A total of 41 mass spectra were obtained. The pressure in the analysis chamber did not exceed 10^8^ mbar during experiments. Peak detection within the mass spectra was performed using SurfaceLab 7 software (ION-TOF, Germany). Ion yields for target peaks were calculated as their normalized intensity relative to the total ion count (TIC) and averaged across all pixels per spectrum. Mass resolution obtained for C_2_H_5_^+^ ion (m/z 29) in positive ion mode and C_3_^−^ ion (m/z 36) in negative ion mode was above 4500 for all measurements.

### Mechanical measurements with AFM

For assessment of the mechanical properties of the cells, the Atomic Force Microscopy (AFM) was used. A Bioscope Resolve AFM (Bruker, Santa Barbara, USA) mounted on an Axio Observer inverted fluorescent microscope (Carl Zeiss, Germany) was equipped with a heated stage, and the sample temperature was kept constant at 37 °C. PeakForce QNM-Live Cell probes (PFQNM-LC-A-CAL, Bruker AFM Probes, Camarillo, CA, USA), short paddle-shaped cantilevers with a pre-calibrated spring constant (the average value of 0.1 N/m) were used, the deflection sensitivity (nm/V) was calibrated from the thermal spectrum using the value of the spring constant^70^. The nanomechanical and topography maps were acquired in the fast force volume (FFV) mode with a map size from 20 × 20 to 80 × 80 μm and from 32 × 32 to 128 × 128 point-measurements. The force curves (F-Z curves) had a vertical ramp distance of 3 μm, a vertical piezo speed of 183 μm/s, and the trigger force of 0.5-1 nN.

The numerical processing of the F-Z curves was done using Python scripts (https://github.com/yu-efremov/ViscoIndent) developed in the previous works^15,16^ with the utilization of the Hertz’s and Ting’s models^71^, for the elastic and viscoelastic processing, respectively. The Young’s modulus (EHertz) with the assumptions of the Hertz’s theory (“apparent” elastic modulus), was calculated from the approach part of the force curves:

1 $$\:F=\frac{4}{3}\frac{E}{(1-{\nu\:}^{2})}\sqrt{R}{\delta\:}^{\frac{3}{2}}$$

where F is the force acting on the cantilever tip; δ is the indentation depth; ν is the Poisson’s ratio of the sample (assumed to be time-independent and equal to 0.5); R is the radius of the indenter; fBEC(δ) is the bottom-effect correction. The latter is a multiplicative, analytically derived correction for indentation contact models that accounts for the finite sample thickness and enables processing of force curves with large indentation depths relative to cell height^15,16^. The multiplicative coefficients were taken from the work^72^. The same curves were processed with the viscoelastic model:

2 $$\:\:F(t,\:\delta\:(t\left)\right)=\frac{4\sqrt{R}}{3(1-{\nu\:}^{2})}{\int\:}_{0}^{{t}_{1}\left(t\right)}\:f\:BEC\left(\delta\:\right)E(t-\xi\:)\frac{d{\delta\:}^{\frac{3}{2}}}{d\epsilon\:}d\xi ;$$

3 $$\:{t}_{1}\left(t\right)=\left\{\begin{array}{c}{t}_{1}\left(t\right)=t,\le\:0\le\:{t}_{m}\\\:{\int\:}_{{t}_{1}\left(t\right)}^{t}\:E(t-\xi\:)\frac{d\delta\:}{d\xi\:}d\xi\:=0,\:t>{t}_{m}\end{array}\right.$$

where t is the time initiated at the contact; tm is the duration of the approach phase; t1 is the auxiliary function determined by Eq. 3; ξ is the dummy time variable required for the integration; and E(t) is the Young’s relaxation modulus for the selected rheology model. Here, we used the power-law rheology (PLR) model (also known as a springpot in parallel with a dashpot)^73^:

4 $$\:E\left(t\right)={E}_{0}{t}^{-\alpha\:}+\eta\:{\delta\:}_{D}\left(t\right)$$

where E0 is the relaxation modulus at t = 1 s (scale factor of the relaxation modulus); α is the power-law exponent; η is the Newtonian viscous term (with Pa*s units); and δD is the Dirac delta function. A larger α value means a larger amount of relaxation; materials exhibit a solid-like behavior at α = 0, and a fluid-like behavior at α = 1. The PLR model described by Eq. (3) was successfully used for the description of the cell mechanics in previous studies^74–76^. The typical indentation depth was 800–1500 nm, examples of the force curves together with the model fits are presented in Fig. S1.

The topography and cell heights were calculated from the force maps based on the contact point position in individual force curves, after detecting the surface level and performing the global tilt correction. Background was then removed by excluding all points with the local height below 200 nm (areas marked with white color on AFM maps). We used the top 50% of each remaining data set over a cell to define the central part, and the lower areas were discarded from the analysis, since the local properties there were highly affected by the high F-actin concentration at the cell periphery. From the cell datasets, the mean arithmetic values of EHertz and viscoelastic parameters were used for the further statistical comparison between the samples.

### Confocal microscopy

For the confocal microscopy, cells were cultured in glass-bottom cell culture dishes (WillCo Wells B.V., Amsterdam, Netherlands). Cells were seeded for 2 days and then fixed in a 4% formaldehyde solution in PBS for 20 min, permeabilized with 0.1% Triton X-100 for 10 min, blocked with 1% bovine serum albumin for 10 min, and stained with Alexa Fluor 488 phalloidin for F-actin and with Hoechst 33,342 for the nuclei (Life Technologies, USA). The samples were washed with PBS and mounted with the SlowFade Diamond Antifade Mountant (Invitrogen, USA). The fluorescent images (Z-stacks with 0.5 μm step) were acquired using an Olympus IX83-FV3000 (Olympus, Japan) confocal laser scanning microscope with a Plan-Apochromat 100x/1.45 N.A. oil immersion objective. The z-stacks are presented as color coded z-projections with respect to the scaling shown in the color scale bar. The orientation of actin filaments in cells was analyzed using the structure tensor method via the ImageJ plugin OrientationJ (v2.0.7)^77^. The coherency parameter reflects the degree of alignment of fibers within a given area (regions of interest [ROIs] covering the majority of each cell). A high coherency value (close to 1) indicates well-aligned fibers, whereas a low value (close to 0) suggests disorganized or randomly oriented (isotropic) fibers. Coherency was quantified for 30 MCF-10 A cells and 61 MCF-7 cells.

### Statistics

The data are presented as the mean values and the standard deviation (SD). To calculate the statistical significance of the differences in microviscosity and ToF-SIMS data, the ANOVA with Bonferroni post-hoc test was used. *p* ≤ 0.05 was considered statistically significant.Principal Component Analysis and Partial Least Squares Discriminant Analysis were used to assess composition differences of mass spectra. For the microviscosity analysis, the number of cells for mean value calculations was 60 in 10 fields of view. For analysis of the AFM data and actin fiber coherency, the non-parametric Mann–Whitney test was performed, the data were collected from at least 20 force volume maps (30 cells) for each cell line.

### Histopathology

Following viscosity imaging experiments, tumors were immediately harvested for histological processing. They were fixed in 10% neutral-buffered formalin, paraffin-embedded, and sectioned at 5 μm thickness. These sections were stained with H&E and visualized under 40x magnification using a Leica DM1000 light microscope.

## Supplementary Information

Below is the link to the electronic supplementary material.

Supplementary Material 1

## Data Availability

The data presented in this study are available on request from the corresponding authors.
