# Supplementary material for: Comparison of viscoelastic properties of breast cancer and normal cells using AFM, FLIM and ToF-SIMS techniques
Source: Sci Rep. 2025 Dec 17;16:1655. doi: 10.1038/s41598-025-31162-3 (PMC12799608; doi:10.1038/s41598-025-31162-3)
Supplement: Supplementary file 1 — Supplementary Material 1 [file 41598_2025_31162_MOESM1_ESM.pdf]

Supplementary Information

Comparison of viscoelastic properties of breast cancer and normal cells using AFM, FLIM and ToF-SIMS techniques

Liubov Shimolina[1*], Yuri M. Efremov[2], Alexander Gulin[3], Nadezhda Ignatova[1], Arseny Aybush[3], Marina K. Kuimova[4], Peter S. Timashev[2], Marina Shirmanova[1]

[1] Institute of Experimental Oncology and Biomedical Technologies, Privolzhsky Research Medical University, Minin and Pozharsky Square, 10/1, Nizhny Novgorod, 603005, Russia
[2]Institute for Regenerative Medicine, Sechenov First Moscow State Medical University (Sechenov University), Trubetskaya str., 8/2, Moscow, 119992, Russia
[3]N.N. Semenov Federal Research Center for Chemical Physics, Russian Academy of Sciences, Kosygina Str., 4, Moscow, 119991, Russia
[4]Department of Chemistry, Imperial College London, White City Campus, London W12 0BZ, United Kingdom
[*]shimolina.l.e@gmail.com

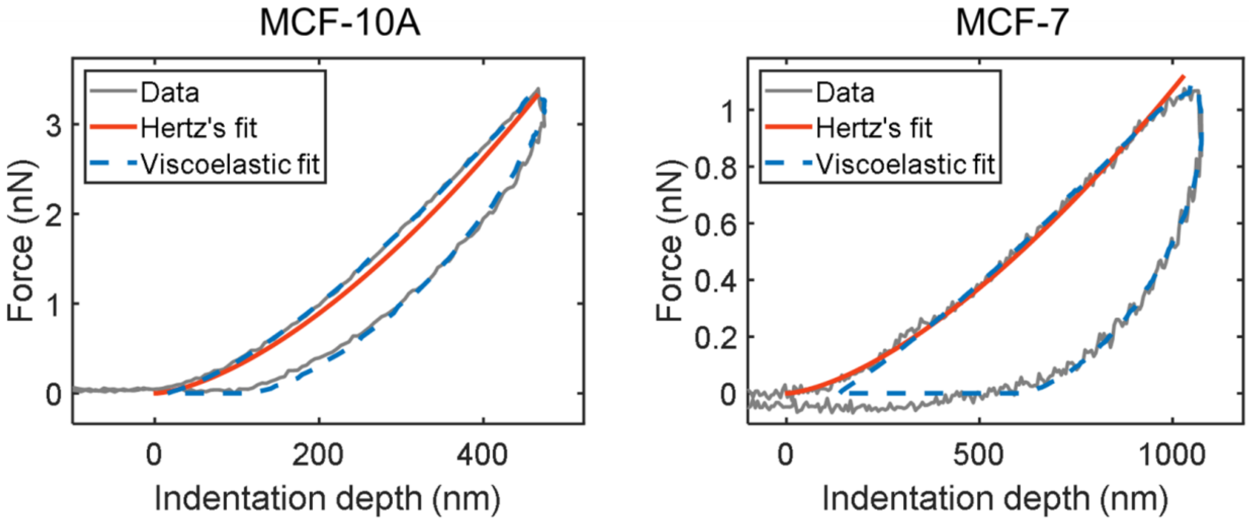

Figure S1. Representative examples of force curves acquired on MCF-10A and MCF-7 cells and processed using the Hertz's and viscoelastic models.

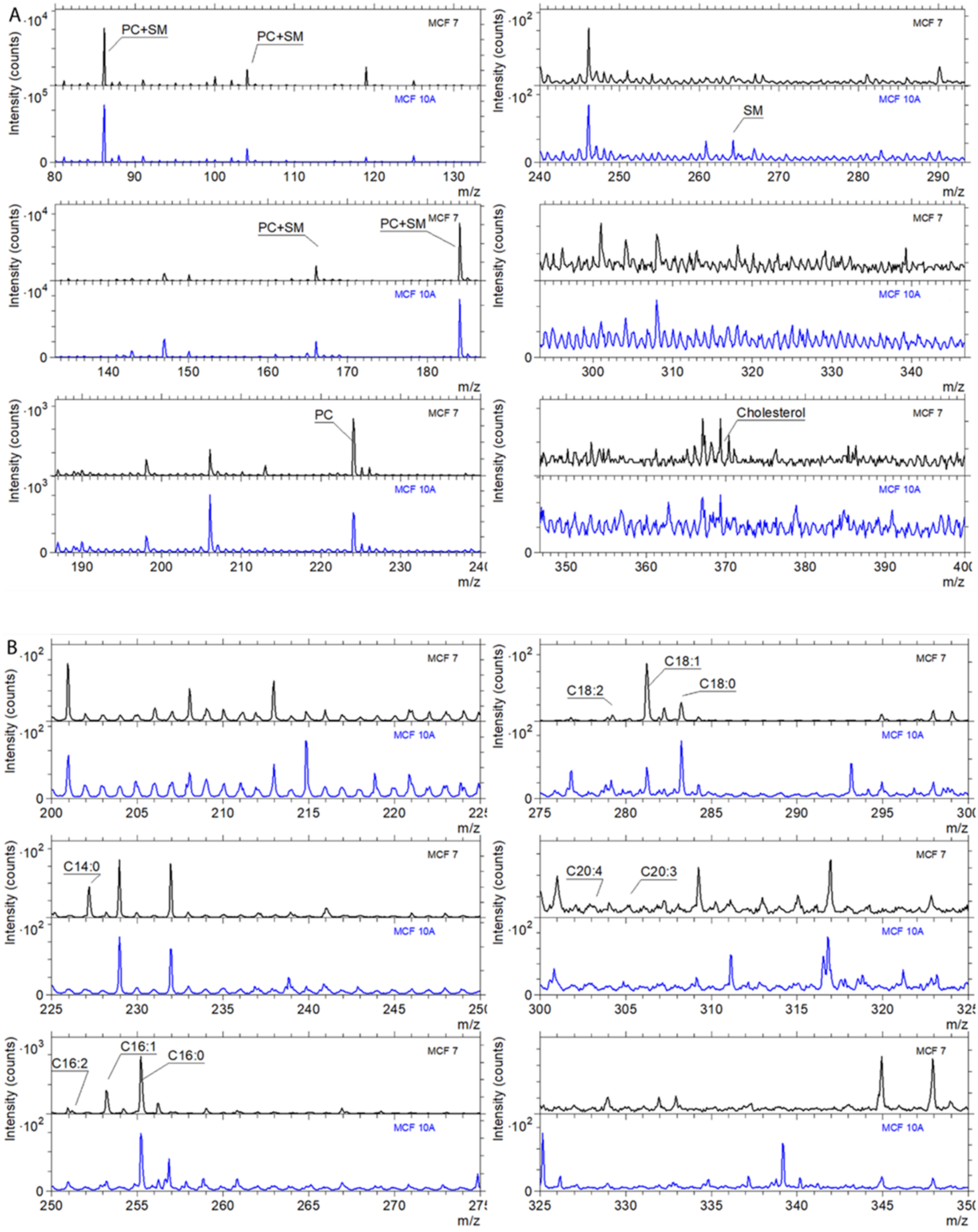

Figure S2. ToF-SIMS mass spectra of normal epithelial MCF-10A (blue) and breast cancer MCF-7 (black) cell lines. (A) Positive ions in the mass range from m/z=80 to m/z=400. (B) Negative ions in the mass range from m/z=200 to m/z=350. PC – phosphatidylcholine, SM – sphingomyelin. Fatty acids are labeled as CX:Y, where X is the chain length and Y is the number of double bonds.

Table S1

A summary table of the parameters

	Parameters	MCF-10A	MCF-7
**The mechanical properties by AFM**	The apparent Young's modulus	↑	↓
	Cell height	↓	↑
	E_0	↑	↓
	η	↑	↓
	α	↓	↑
	Coherency (Alignment of actin filaments)	↑	↓
**The membrane microviscosity by**	Membrane microviscosity in vitro	↓	↑
	Membrane microviscosity in vivo	↓	↑
**The membrane's lipid composition by ToF-SIMS**	PI	↑	↓
	PC	↑	↓
	SM	↓	↑
	Chol	=	=
	MonoFA	↓	↑
	PolyFA	↑	↓
	Tyr	↓	↑
	C14:0	↓	↑
	C18:1	↓	↑
	C18:0	↓	↑
